# Factors Affecting the Occurrence of Mental Health Problems in Female Cancer Survivors: A Nationwide Cohort Study

**DOI:** 10.3390/ijerph19148615

**Published:** 2022-07-15

**Authors:** So Young Kim, Yeonju Lee, Sang Baek Koh

**Affiliations:** 1 Department of Preventive Medicine, Yonsei University Wonju College of Medicine, Wonju 26426, Korea; atom7010@gmail.com; 2 Health Insurance Research Institute, National Health Insurance Service, Wonju 26464, Korea; iyeonst@gmail.com

**Keywords:** female cancer survivors, mental health, health behaviors

## Abstract

The purpose of this study is to determine the effect of cancer survivorship stage and health-related behaviors on the risk of developing mental health problems (depressive and anxiety disorders) in women who have experienced cancers that affect women (breast cancer, cervical cancer, ovarian cancer, endometrial cancer). Using the healthcare utilization and medical checkup data from 2010 to 2020 provided by the National Health Insurance Service, the occurrence of mental health problems since 2020 was tracked for 36,801 women diagnosed with cancer. The occurrence of mental health problems was defined as the cases in which the disease code was assigned to anxiety disorders (F40~F44, F48) and depressive disorders (F32~34, F41.2, F92) as presented in ICD-10. To evaluate the effect of cancer survivorship stage and health-related behaviors on the development of mental health problems, the hazard ratio (HR) and 95% confidence intervals (CI) were calculated using the Cox proportional hazard model. During the follow-up period of 5.6 years, anxiety disorder occurred in 14,698 (39.9%), and by cancer type, breast cancer survivors accounted for the most at 1.02 per 1000 person-years. The risk of anxiety disorders increased in those who experienced cervical cancer (AHR, 1.08, 95% CI, 1.03–1.13) and those in the acute survivorship stage (AHR, 1.38, 95% CI, 1.22–1.55). In terms of health-related behaviors, the risk of developing anxiety disorder was shown to be reduced in drinkers (AHR, 0.91, 95% CI, 0.87–0.96), former smokers (AHR, 0.86, 95% CI, 0.77–0.97), and current smokers (AHR, 0.80, 95% CI, 0.71–0.90). During the follow-up period of 5.6 years, depressive disorder occurred in 6168(16.8%), and by cancer type, ovarian cancer survivors accounted for the most at 1.30 per 1000 person-years. The risk of developing depressive disorders was highest in those who experienced ovarian cancer (AHR, 1.40, 95% CI, 1.27–1.53) and those in the acute survivorship stage (AHR, 2.99, 95% CI, 2.60–3.42). For health-related behaviors, the risk of developing depressive disorders was increased in former smokers (AHR, 1.32, 95% CI, 1.14–1.54), current smokers (AHR, 1.21, 95% CI, 1.04–1.41), and those with insufficient physical activity (AHR, 1.09, 95% CI, 1.02–1.15). It has been confirmed that cancer type, cancer survivorship stage, and health-related behaviors, such as smoking, drinking, and physical activity, are significantly related to the risk of mental health problems. Thus, it is necessary to develop strategies to cope with mental health problems at the individual and national levels and to develop interventions to promote a more active lifestyle.

## 1. Introduction

According to the 2020 National Cancer Registration Statistics in Korea, the 5-year relative survival rate for people who have experienced cancer in the past five years is 66.5%, and females have a higher survival rate (71.5%) than males (62.4%). The increase in the relative survival rate leads to an increase in the number of long-term cancer survivors. The number of survivors of long-term cancer over a duration of 5 years or more is approximately 1.3 million, accounting for about 60% of the total cancer survivors. In addition, the proportion of female cancer survivors who have experienced cancers that affect women, including breast cancer, accounts for more than 30% of all cancer survivors [1]. Thus, the importance of health management for the increasing number of female cancer survivors is being emphasized. 

Those who have experienced cancer encounter physical and psychological pain resulting from the side effects of surgery, radiation, and chemotherapy from the time of diagnosis. Even after the end of treatment, they experience feelings of helplessness, depression, and anxiety in the process of adjusting to life at home and work due to recurrence, secondary cancer, and altered physical functioning [2]. When women are diagnosed with gynecological cancer in particular, they can experience more mental health problems than men because of their femininity-associated factors [3]. Previous studies have also shown that gynecological cancer survivors had the highest level of anxiety compared to survivors of other cancers [4], and breast cancer survivors had the highest depression score compared to those who experienced other cancers [5]. Psychological pain such as depression and anxiety in female cancer survivors can lead to reduced quality of life [2], lower treatment compliance [6], and lower survival rates [7,8]; therefore, it must be monitored and managed promptly.

Mullan (1895) described the stages of cancer survivorship by applying the experience of cancer survivors to the traditional concept of cancer survivorship, which defines survivors as those who survive for more than 5 years without evidence of recurrence or metastasis after treatment. Mullan classified the stage at which the patients receive various treatments for less than 2 years since diagnosis as the acute stage, the period between 2 to 5 years after diagnosis in which the disease remission or follow-up after active treatment is completed as the extended stage, and the stage in which cancer cell activity almost disappears after more than 5 years after diagnosis as the permanent stage [9]. Previous studies revealed that health-related behaviors differ according to these stages of survivorship [9], and inappropriate health-related behaviors increase as the duration of the disease increases [10,11] Health-related behaviors such as smoking, drinking alcohol, and physical activity are defined by the International Agency for Research on Cancer (IARC) as carcinogenic factors for gynecological cancers such as ovarian and uterine cancers, and breast cancers [12]. Inappropriate health-related behaviors have been reported to affect the occurrence of mental health problems [13,14,15]. 

A previous study revealed that smokers who experienced cancer reported severe psychological distress more frequently due to depression and anxiety compared to nonsmokers who experienced cancer [16], and the higher the depression score, the higher the risk of drinking in breast cancer survivors [15]. In addition, physical activity promotes the release and synthesis of serotonin as well as improvements of physical symptoms such as fatigue and pain, thereby reducing anxiety and depressive symptoms [17]. The mental health scores of cancer survivors who did not follow the physical activity guidelines were found to be lower than that of those who did [14]. Consequently, it is necessary to examine the relationship between health-related behaviors such as smoking and physical activity and the mental health status of female cancer survivors. Most previous studies on mental health problems of women who have experienced cancer focused on breast cancer [15,18,19], health-related behavior, and quality of life at a certain point in time [2,9,10,17] and identified the relationship over a short-term survival period of fewer than five years [3,20,21]. However, there are insufficient studies on survivors of other female cancers, such as ovarian and endometrial cancers, which have a high incidence rate as well as high survival and recurrence rates. Therefore, by establishing a national population-based female cancer cohort over the past 10 years, this study investigated the hypothesis that cancer type, cancer survivorship stage, and health-related behaviors are related to the risk of mental illness among women who have experienced breast, cervical, ovarian, endometrial, and cervical cancers.

### Purpose of Study

The purpose of this study is to investigate the effect of cancer survivorship stage and health-related behaviors on the risk of mental health problems (depressive and anxiety disorders) in women who have experienced cancers that affect women (breast cancer, cervical cancer, ovarian cancer, and endometrial cancer). The specific research objectives are as follows:

1. Identifying general and clinical characteristics of study participants;
2. Identifying the incidence and risk of anxiety disorders according to the cancer survivorship stage and health-related behaviors of the study participants;
3. Identifying the incidence and risk of depressive disorders according to the cancer survivorship stage and health-related behaviors of the study participants.

## 2. Materials and Methods

### 2.1. Research Design

This study is a retrospective cohort study with secondary data analysis to determine the relationship between survivorship stage and health-related behaviors and the occurrence of mental health problems in women who have experienced cancer (breast cancer, cervical cancer, ovarian cancer, endometrial cancer) in Korea.

### 2.2. Data Source

This study utilized the National Health Insurance Service’s insurance qualification, claim and health checkup data from 2009 to 2020. The National Health Insurance Service is the sole insurer in Korea, with 97% of the population enrolled in health insurance and the remaining 3% in medical benefits. A single database with integrated health insurance and medical benefits insurance was constructed in 2006, and a qualification database (age, gender, income, region, qualification type, etc.), a claim database (consultation statements, diagnosis statements defined by the International Classification of Disease 10th revision (ICD-10), prescription statements, outpatient visits, and hospitalization records, etc.), a health checkup database (medical checkup results), death information, and medical institution information, etc., have been established as sub-databases [22]. Furthermore, the National Health Insurance Service carries out standardized health checkups for its members for the purpose of early detection of diseases. All members are required to receive a health checkup every two years at an institution recognized by the National Health Insurance Service, and the checkup includes physical measurements (e.g., height and weight), clinical examinations (e.g., blood pressure, fasting blood sugar, blood lipid tests), and questionnaires (e.g., health-related behaviors such as smoking, drinking, physical activities, and medical history). Physical measurements and clinical examinations are conducted by the staff at the medical institution, and examinees’ self-response is recorded in the questionnaires. 

### 2.3. Study Participants and Data Collection Process

In this study, female cancer survivors, cancer survivorship stage, and mental health problems were defined. A cancer survivor is defined as a case in which one of the codes for breast cancer (C50), cervical cancer (C53), ovarian cancer (C56), and endometrial cancer (C54.1) presented in the International Classification of Disease 10th revision (ICD-10) is assigned in addition to the special code for cancer (V193). The cancer survivorship stage refers to the acute, extended, and permanent stages as defined by Mullan [9]. The acute stage refers to the stage less than two years from diagnosis, the extended stage refers to the period between two to five years after diagnosis, and the permanent stage refers to the stage more than five years after diagnosis. The date of diagnosis was based on the date on which the special code for cancer (V193) in the program implemented by NHIS to enhance the health coverage for severe disease was assigned. Mental health problems were defined as cases in which the codes for anxiety disorders (F40~F44, F48) and depressive disorders (F32~34, F41.2, F92) presented in the International Classification of Disease 10th revision (ICD-10) were assigned. 

**Figure 1:**
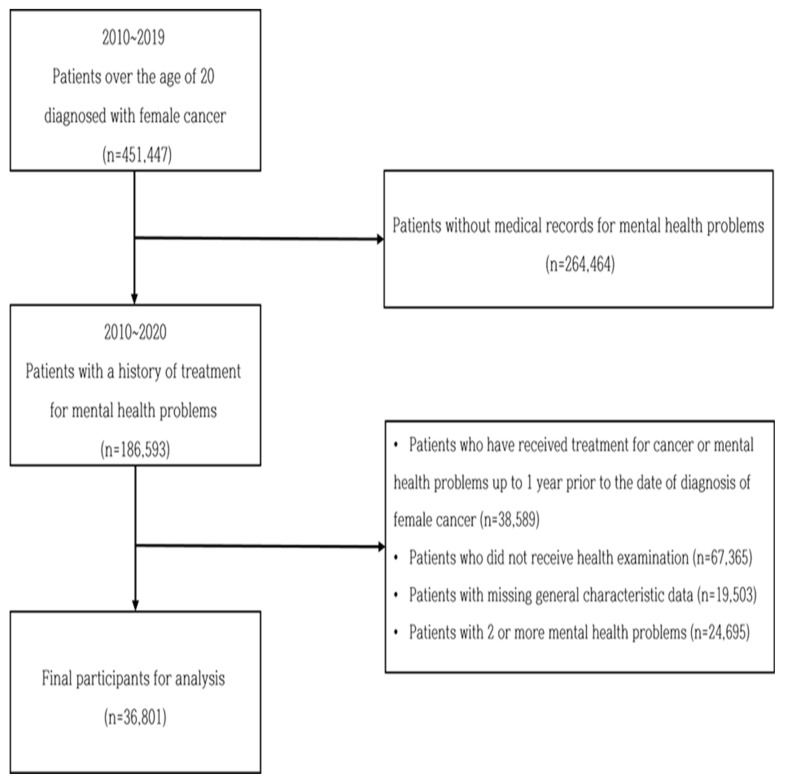
Flow chart of study participants.

The participants’ selection process in this study is shown in Figure 1. First, to establish a cohort of women who had experienced cancer, women over the age of 20 whose main disease in the health insurance healthcare utilization history were female cancer, including breast cancer (C50), cervical cancer (C53), ovarian cancer (C56), or endometrial cancer (C54.1), who were also assigned the special code (V193) for health coverage for a severe disease at the time of diagnosis from 1 January 2010, to 31 December 2019, were extracted. Among them, women with a history of medical utilization due to mental health problems were extracted. The period between the date on which the special code (V193) was assigned and one year before the very date was set as the wash-out period. Patients with mental health problems in this wash-out period were excluded; therefore, only those with a history of healthcare utilization for mental health problems after the initial cancer diagnosis were selected. Among them, patients whose main disease in the health insurance healthcare utilization history included anxiety disorders (F40~F44, F48) and depressive disorders (F32~34, F41.2, F92) were extracted. Finally, a total of 36,801 were selected as study participants, excluding those who did not undergo health checkups within 1 year of the occurrence of anxiety or depressive disorders to confirm the effect of health-related behaviors on the occurrence of mental health problems.

### 2.4. Measurement

#### 2.4.1. General Characteristics

Age, insurance premium quartiles from qualification data, cancer type derived from claim data, blood pressure, body mass index from the health checkup data, and blood test results were the participants’ characteristics considered in this study. 

Blood pressure was recorded as systolic blood pressure (mmHg) and diastolic blood pressure (mmHg). Body mass index (BMI) was calculated by dividing the measured weight by the square of the height (weight (kg)/height^2^ (m^2^)) and classified into the underweight group (BMI < 18.5 kg/m^2^), normal group (18.5 ≤ BMI < 23.0 kg/m^2^), overweight group (23.0 ≤ BMI < 25.0 kg/m^2^), class 1 obesity group (25.0 ≤ BMI < 30.0 kg/m^2^), and class 2 to severe obesity group (BMI ≥ 30.0 kg/m^2^) according to the criteria presented in the 2020 Obesity Treatment Guidelines [23]. 

For blood test results, blood lipids (total cholesterol, HDL cholesterol, LDL cholesterol, triglycerides) and fasting blood sugar were considered. According to the 2018 dyslipidemia treatment guidelines [24], total cholesterol (TC) was categorized as normal (TC < 200 mg/dL), borderline (200 ≤ TC < 240 mg/dL), and high (TC ≥ 240 mg/dL). High-density lipoprotein cholesterol (HDL) was categorized as low (HDL < 40 mg/dL) and average (HDL ≥ 40 mg/dL). Low-density lipoprotein cholesterol (LDL) was categorized as normal (LDL < 130 mg/dL), borderline (130 ≤ LDL < 160 mg/dL), and high (LDL ≥ 160 mg/dL). Triglycerides (TG) were categorized as average (TG < 150 mg/dL), borderline (150 ≤ TG < 200 mg/dL), and high (TG ≥ 200 mg/dL). Furthermore, fasting blood sugar (FBS) test results were categorized as average (FBS < 100 mg/dL), fasting blood sugar disorder (100 ≤ FBS < 125 mg/dL), and diabetic (FBS ≥ 126 mg/dL) according to the 7th edition of the 2021 Diabetes Treatment Guidelines [25].

#### 2.4.2. Survivorship Stage

The survivorship stage is defined as acute if it has been less than two years from the date of diagnosis, extended if it has been between two to five years from the date of diagnosis, and permanent if more than five years had elapsed from the date of diagnosis, where the date of diagnosis is based on the date on which the special code for cancer (V193) was assigned. 

#### 2.4.3. Health-Related Behaviors

For health-related behaviors, the questionnaire results for smoking status, drinking status, and physical activity surveyed during the health checkup were used. 

Based on the answer to the question, “Have you ever smoked more than 100 cigarettes in your life?”, participants were classified as nonsmokers if they answered “No,” as a former smoker if they answered “Yes, but I have quit,” and as current smokers if they answered “Yes, and I am currently smoking.” Drinking status was classified into nondrinkers, moderate-risk drinkers, and high-risk drinkers. Participants were classified as nondrinkers if they answered “0” to the question “How many days per week do you drink on average?” or answered “None,” to the question, “How many times have you drank alcohol in the past year?” According to the amount of alcohol converted into a standard glass, moderate-risk drinkers and high-risk drinkers were classified as drinkers. Alcohol consumption using a standard drink is determined by the number of drinks according to age. Those over the age of 65 are considered moderate-risk drinkers if they drink less than four glasses, and those under the age of 65 are considered moderate-risk drinkers if they drink less than eight glasses. Anyone who exceeds the standards for moderate-risk drinking is considered a high-risk drinker. Lastly, for the physical activity, the physical activity of 150 min in terms of time or 5 or more times a week in terms of frequency from the results of the questionnaire was defined as average or health-promoting, and anything below was defined as insufficient physical activity. 

#### 2.4.4. Study Outcome: Mental Health Case Ascertainment

For the selected study participants, they were confirmed to have a diagnosis of mental illness if anxiety disorders (F40~F44, F48) and depressive disorders (F32~34, F41.2, F92) were identified as the main disease in the health insurance healthcare claim data from the time of diagnosis for cancer to the end of the follow-up period.

### 2.5. Ethical Consideration

This study was approved for IRB review exemption (IRB No, CR321342) by the institutional ethics review committee of Yonsei University Wonju Severance Christian Hospital, and it was analyzed using de-identified data.

### 2.6. Statistical Analysis

Analysis in this study was performed using SAS v.9.4. (SAS Institute Inc., Cary, NC, USA). Among the general and clinical characteristics of study participants, frequency and percentage were analyzed for categorical variables, and mean and standard deviation were analyzed for continuous variables. The incidence rate of mental health problems was presented as the number of events per 1000 person-years, and the hazard ratio (HR) and 95% confidence intervals (CI) were calculated using the Cox proportional2 hazard model.

## 3. Results

### 3.1. General Characteristics of Study Participants

The general characteristics of the study participants are shown in Table 1. The average age of the participants at the time of diagnosis was 56.6 years (SD 10.3 years), and in the age group distribution, the 50~59 group accounted for the most at 37.6%. By cancer survivorship stage, the permanent stage accounted for the most at 77.6%, followed by the extended stage at 19.2%, and the acute stage at 3.3%. Among the diagnosed cancer type, breast cancer was the most common at 74.6%, and endometrial cancer was the least common at 5.8%. As for medical history, hypertension was the most prevalent at 23.3%, followed by diabetes at 8.7%, and heart disease at 2.4%. In terms of health-related behaviors, current smokers accounted for 2.5% and drinkers for 17.4% of the participants, and 73.3% showed insufficient physical activity. In terms of body mass index, the normal group showed the largest distribution at 41.8%, but 55% were overweight, including obesity and severe obesity. The blood lipid test results showed that 46.2% had borderline or high levels of total cholesterol, 33.2% had borderline or high levels of low-density lipoprotein cholesterol, and 23.6% had borderline or high levels of triglycerides. Furthermore, 92.6% had high levels of high-density lipoprotein cholesterol, and 34.7% had a fasting blood sugar level that corresponds to impaired fasting glucose or diabetes. 

### 3.2. Incidence Rate and Risk of Mental Health Problems According to Cancer Survivorship Stage and Health-Related Behavior

#### 3.2.1. Incidence Rate of Mental Health Problems

The median follow-up period for the occurrence of anxiety disorders was 5.6 years. During the follow-up period, anxiety disorders were newly developed in 14,698 (39.9%) patients, and the incidence rate of anxiety disorders was 0.95 cases per 1000 person-years. By cancer type, breast cancer had the highest incidence rate at 1.02 cases, and cervical cancer had the lowest at 0.78 cases. By survivorship stage, the acute stage had the highest incidence rate at 5.53 cases, and the permanent stage had the lowest incidence rate at 0.86 cases <Table 2>. 

The median follow-up period for the occurrence of depressive disorders was 7.0 years. During the follow-up period, depressive disorders were newly developed in 6168 (16.8%) patients, and the incidence rate of depressive disorders was 1.08 cases per 1000 person-years. By cancer type, ovarian cancer had the highest incidence rate at 1.30 cases, and cervical cancer had the lowest at 0.88. By cancer survivorship stage, the acute stage had the highest incidence at 5.40 cases, and the permanent stage had the lowest incidence rate at 0.86 <Table 3>.

#### 3.2.2. Risk of Mental Health Problems

The analysis results for the risk of developing anxiety disorders are shown in Table 2. Looking at the results by cancer type, the risk of developing anxiety disorders in cervical cancer survivors was 1.09 times higher (HR 1.09, 95% CI 1.04–1.14) compared to breast cancer survivors, whereas it was 0.85 times lower (HR 0.85, 95% CI 0.79–0.91) in ovarian cancer survivors. 

Looking at the results by survivorship stage, the risk of developing anxiety disorders in the acute stage was 1.3 times higher (HR 1.38, 95% CI 1.22–1.55) compared to the permanent stage, whereas it was 0.93 times lower (HR 0.93, 95% CI 0.88–0.98) in the extended stage.

Looking at the results by health-related behaviors, the risk of developing anxiety disorders based on smoking status was 0.86 times lower (HR 0.86, 95% CI 0.77–0.97) in former smokers and 0.80 times lower (HR 0.80, 95% CI 0.71–0.90) in current smokers compared to nonsmokers. The risk of developing anxiety disorders based on drinking status was 0.91 times lower (HR 0.91, 95% CI 0.87–0.96) in drinkers compared to nondrinkers. The hazard ratio of anxiety disorders according to physical activity was not statically significant in all models. 

The analysis results for the risk of developing depressive disorders are shown in Table 3. Looking at the results by cancer type, the risk of developing depressive disorders was 1.40 times higher (HR 1.40, 95% CI 1.27–1.53) in ovarian cancer survivors compared to breast cancer survivors, followed by 1.18 times (HR 1.18, 95% CI 1.07–1.31) in endometrial cancer survivors and 1.14 times (HR 1.14, 95% CI 1.06–1.23) in cervical cancer survivors. 

Looking at the results by survivorship stage, the risk of developing depressive disorders was 2.99 times higher (HR 2.99, 95% CI 2.60–3.42) in the acute stage and 2.14 times higher (HR 2.14, 95% CI 2.00–2.28) in the extended stage compared to the permanent stage.

Looking at the results by health-related behaviors, the risk of developing depressive disorders based on smoking status was 1.32 higher (HR 1.32, 95% CI 1.14–1.54) in former smokers and 1.21 times higher (HR 1.21, 95% CI 1.04–1.41) in current smokers compared to nonsmokers, The risk of developing depressive disorders based on drinking status was 0.89 times lower (HR 0.89, 95% CI 0.82–0.95) in drinkers compared to nondrinkers in the overall adjusted model. The hazard ratio of depressive disorders according to physical activity was 1.09 times higher (HR 1.09, 95% CI 1.02–1.15) in the insufficient physical activity group compared to the average or health-promoting group.

## 4. Discussion

This study investigated whether cancer survivorship stage and health-related behaviors affect the occurrence of mental health problems (depressive and anxiety disorders) for women who have experienced cancer (breast cancer, cervical cancer, ovarian cancer, endometrial cancer) and attempted to provide basic data to improve their mental health and quality of life. As a result, it was confirmed that cancer type, survivorship stage, and health-related behaviors were related to the risk of developing mental health problems.

By cancer type, the risk of developing anxiety disorders was shown to increase in those who experienced cervical cancer compared to breast cancer. The risk of developing depressive disorders was found to increase in the order of those who experienced ovarian cancer, endometrial cancer, and cervical cancer compared to breast cancer. No studies compared the risk of depressive and anxiety disorders among women with the same cancer types as in this study, so it was not possible to directly compare the study results. Yet, a study comparing the prevalence rate of depressive symptoms among women who had experienced cervical cancer, ovarian cancer, and uterine cancer compared to women without cancer reported that women who had cancer had a significantly higher prevalence of depression than those without cancer, and the prevalence of depression was significantly higher in the order of those who experienced ovarian cancer, cervical cancer, and uterine cancer [26]. Ovarian cancer is often diagnosed later than other female cancers, so it is difficult to treat and has a high recurrence and mortality rate. Furthermore, hysterectomy including bilateral ovaries to treat cancer causes premature menopause and loss of fertility in premenopausal women, thereby promoting the risk of depressive disorders due to the changes in hormonal status [27]. In addition, during the treatment process, loss of body parts due to uterine and appendage extraction, wounds due to surgery, chemotherapy, radiation therapy, etc., may have a significant impact on the occurrence of anxiety and depressive disorders. Thus, more attention should be paid to the potential damage to the participant’s body when deciding on a treatment method according to cancer diagnosis, and a customized cancer management intervention should be utilized considering the age group of the participant undergoing organ loss.

By survivorship stage, the risk of developing depressive disorders was increased in the acute and extended stages compared to the permanent stage. The risk of developing anxiety disorders was increased in the acute stage but appeared to decrease in the extended stage. This is consistent with the results of previous studies [28], which reported that negative emotional reactions such as anger, depression, anxiety, fear, and sadness are experienced more in the acute stage than in other stages due to cancer diagnosis, treatments including surgery, chemotherapy, radiation, and side effects from the treatment process, and the patients experience increased emotional distress in the extended stage, the stage at which treatment is completed, due to uncertainty related to recurrence or secondary cancer than when receiving treatment [29]. These results suggest that cancer survivors require continuous mental health management from the time they are diagnosed through the entire treatment period. 

By smoking status, former and current smokers were found to have an increased risk of developing depressive disorders compared to nonsmokers. This was consistent with the results of a previous study in which the history of depressive disorders significantly increased with smoking history [13]. On the other hand, the risk of anxiety disorders was reduced in former and current smokers compared to nonsmokers. This opposes the results of a study that analyzed the factors related to anxiety disorders during the first year after a cancer diagnosis, which reported that smokers had an increased probability of developing anxiety disorders compared to nonsmokers [30]. This discrepancy may be attributed to the fact that the previous study targeted cancer survivors who survived up to 1 year after a cancer diagnosis, whereas 77.6% of the participants in this study were in the permanent survivorship stage, surviving more than 5 years after diagnosis. Therefore, as the survival period increased, it is thought that the participants failed to maintain correct health behaviors and selected smoking to reduce anxiety. This suggests that it is necessary to develop an effective and sustainable intervention program that can healthily reduce depression and anxiety for cancer survivors in the future. 

By drinking status, the risk of developing anxiety and depressive disorders was shown to be reduced in drinkers compared to nondrinkers. Previous research also found that in women, light (3 drinks or less/week) or moderate (7 drinks or less/week) drinkers were less likely to be depressed than nondrinkers and heavy (7 drinkers or more/week) drinkers [31], and a meta-analysis also showed that light (<84 g/week) or moderate (85–168 g/week) drinkers had a lower risk of depression compared to nondrinkers [32]. These results can be explained by the coping model and the physiological effects of smoking and drinking. The coping model suggests that people engage in behaviors such as smoking and drinking as a mechanism to cope with negative emotions such as anxiety, depression, and pain [33]. When smoking, the nicotine contained in cigarettes affects the excessive or abnormal release of dopamine by neurons, and the temporary positive emotions experienced under the influence of nicotine alleviate anxiety. Thus, people continue to smoke to maintain these positive emotions [13]. Furthermore, drinking has an inhibitory effect on the central nervous system and enhances the action of gamma-aminobutyric acid (GABA), an inhibitory neurotransmitter. These neurotransmitters temporarily relieve anxiety and depression by inducing a relaxing effect to release tension and causes people to continue drinking to maintain these sensations. However, continuous exposure to nicotine and alcohol, which can induce dependence, can cause the mechanism of dopamine synthesis to be disrupted and the secretion of serotonin to be decreased, which rather acts as a factor inducing depression [34].

Recently, with the increase in the long-term survival rate of cancer-experienced patients, maintaining appropriate health-related behaviors is being emphasized, but it is difficult to continuously maintain high motivation for self-management at the time of diagnosis to follow appropriate health-related behaviors. Smoking and drinking are carcinogens defined by the International Agency for Research on Cancer (IARC) [12] and may cause not only mental health problems but also exacerbation of primary cancers and the occurrence of secondary cancers. Continuous efforts are necessary to monitor the health-related behaviors of patients with cancer from the time intensive clinical treatment is completed and to maintain appropriate health-related behaviors.

Lastly, in terms of physical activity, the group with insufficient physical activity showed an increased risk of developing depressive disorders compared to the group with average or health-promoting physical activity. Previous research also found that those who did not follow the guidelines for physical activity had lower mental health scores than those who did [14]. In general, physical activity promotes the release and synthesis of serotonin and can affect the reduction of depressive symptoms. Thus, it is thought that physical activity will help reduce depression in patients who have experienced cancer. In addition, according to the healthcare guideline for cancer survivors [35], it is suggested that physical activity in cancer patients not only helps improve physical symptoms such as fatigue and pain and mental symptoms such as depression, anxiety, and sleep disturbance but also ultimately helps improve the quality of life. Physical activity should be emphasized as a low-cost non-pharmaceutical intervention. 

This study has several limitations. First, since most of the existing studies used hospital data which limited the research process, this study used claim data from the National Health Insurance Service for more representative results. However, the diagnosis code of healthcare utilization data is not as accurate as a diagnosis obtained from a structured clinical interview using questionnaires; therefore, there is a possibility of a misdiagnosis. Second, health-related behaviors such as drinking, smoking, and physical activity were investigated in a subjective self-report method and may have been overreported or underreported. Thus, caution is required in interpreting the study results. Third, information on the clinical stage of cancer, which can explain the post-diagnosis behavior, is missing from the health insurance claim data used for analysis. Therefore, it is necessary to consider the severity at the time of diagnosis to accurately identify a patient’s medical behavior.

Despite these limitations, this study is significant because it included those who had long-term cancer experiences for a long period from immediately after diagnosis up to 10 years using national representative data and was able to determine the difference in the risk of anxiety and depressive disorders according to the survivorship stage and health-related behaviors of women who experienced cancer. It can be used as a basis for policy decisions to improve the mental health of female cancer patients in the future.

## 5. Conclusions

Based on the insurance qualification, claim, and health checkup data provided by the National Health Insurance Service, which are national data, this study investigated the effects of cancer survivorship stage and health-related behaviors on the risk of developing mental health problems in women who have experienced cancer using the Cox proportional hazard model to examine the hazard ratio. As a result, it was confirmed that the risk of developing anxiety disorders increased in those who experienced cervical cancer compared to breast cancer, and the risk of depressive disorders increased in the order of those who experienced ovarian cancer, endometrial cancer, and cervical cancer compared to breast cancer. By cancer survivorship stage, the risk of developing depressive disorders was confirmed to increase in the acute and extended stages, and the risk of developing anxiety disorders was shown to increase in the acute stage and decrease in the extended stage. By smoking status, former and current smokers had an increased risk of developing depressive disorders but decreased risk of developing anxiety disorders compared to nonsmokers. By drinking status, the risk of developing anxiety and depressive disorders was reduced in drinkers compared to nondrinkers. The risk of developing depressive disorders increased in the group with insufficient physical activity compared to the average or health-promoting group in terms of physical activity.

Most of the medical staff caring for cancer patients are focused on cancer treatment, so the interest in mental health is relatively small. Medical staff in charge of treating cancer survivors must be aware that mental health problems can occur depending on the cancer type and survival stage and promote cancer survivors’ participation in various mental health programs.

Based on these results, we would like to make the following recommendations. 

First, a large-scale study that can improve mental health and factors that can affect the occurrence of mental health problems in not only female cancer survivors but also male cancer survivors is needed. Second, it is necessary to prepare a national roadmap for the mental health promotion programs provided by various multidisciplinary experts such as oncologists, psychiatrists, nurses, and psychotherapists. Third, it is necessary to prepare additional support benefits to promote cancer survivors’ participation in mental health promotion programs.

## Figures and Tables

**Table 1:** General characteristics of study participants (*n* = 36,801).

Variable		Category	*n*	%	Mean ± SD
Age (years) (at cancer diagnosis)					56.6 ± 10.3
		<30	126	0.3	
		30~39	1657	4.5	
		40~49	10,715	29.1	
		50~59	13,826	37.6	
		60~69	7615	20.7	
		≥70	2862	7.8	
Survival stage		Acute SS	1198	3.3	
		Extended SS	7052	19.2	
		Permanent SS	28,551	77.6	
Cancer type		Breast	27,464	74.6	
		Cervical	4873	13.2	
		Ovarian	2329	6.3	
		Endometrial	2135	5.8	
History	Diabetes	Yes	3188	8.7	
	Hypertension	Yes	8573	23.3	
	Heart disease	Yes	868	2.4	
Health-related behavior	Smoking	Nonsmoker	35,012	95.1	
		Former smoker	886	2.4	
		Current smoker	903	2.5	
	Drinking	Nondrinker	30,382	82.6	
		Drinker	6419	17.4	
	Physical activity	Insufficient	26,988	73.3	
		Average or health-promoting	9813	26.7	
BMI (Kg/m^2^)		<18.5	1155	3.1	23.8 ± 3.4
		18.5~22.9	15,397	41.8	
		23~24.9	8705	23.7	
		25~29.9	9899	26.9	
		≥30	1645	4.5	
Systolic BP (mmHg)					121.4 ± 15.5
Diastolic BP (mmHg)					74.9 ± 9.9
Fasting blood glucose (mg/dL)		<100	24,028	65.3	
		100~125	10,211	27.7	
		≥126	2562	7.0	
Total cholesterol (mg/dL)		<200	19,789	53.8	
		200~239	11,96	32.5	
		≥240	5048	13.7	
High-density lipoprotein (HDL) cholesterol (mg/dL)		<40	2726	7.4	
		≥40	34,075	92.6	
Low-density lipoprotein (LDL) cholesterol (mg/dL)		<130	24,567	66.8	
		130~159	8120	22.1	
		≥160	4114	11.2	
Triglycerides (mg/dL)		<150	28,128	76.4	
		150~199	4769	13.0	
		≥200	3904	10.6	

SS = survivorship stage.

**Table 2:** Hazard ratios and 95% confidence intervals of anxiety disorders based on cancer survivorship stage and health behaviors.

Variables			Participants (*n*)	Events (*n*)	Follow-Up Duration (Person-Years)	Incidence Rate (Per 1000 Person-Years)	Model 1				Model 2				Model 3			
							HR	95% CI		*p*-Value	HR	95% CI		*p*-Value	HR	95% CI		*p*-Value
All Cause Anxiety Disorders			36,801	14,698	15,402,585	0.95												
Cancer type		Breast	27,464	10,689	10,500,933	1.02	1 (ref.)				1 (ref.)				1 (ref.)			
		Cervical	4873	2280	2,932,235	0.78	1.09	1.04	1.14	0.00	1.09	1.04	1.14	0.00	1.08	1.03	1.13	0.00
		Ovarian	2329	816	927,158	0.88	0.85	0.79	0.91	<0.0001	0.85	0.79	0.91	<0.0001	0.85	0.79	0.91	<0.0001
		Endometrial	2135	913	1042,259	0.88	1.06	0.99	1.13	0.10	1.06	0.99	1.13	0.10	1.06	0.99	1.14	0.09
SS		Acute SS	1198	276	49,907	5.53	1.42	1.26	1.60	<0.0001					1.38	1.22	1.55	<0.0001
		Extended SS	7052	1761	643,081	2.74	0.94	0.89	0.99	0.02					0.93	0.88	0.98	0.00
		Permanent SS	28,551	12,661	14,709,597	0.86	1 (ref.)								1 (ref.)			
Health -related behavior	Smoking	Nonsmoker	35,012	14,121	14,845,414	0.95	1 (ref.)				1 (ref.)							
		Former smoker	886	296	305,749	0.97	0.86	0.77	0.97	0.01	0.86	0.77	0.97	0.01				
		Current smoker	903	281	251,422	1.12	0.80	0.71	0.90	0.00	0.80	0.71	0.90	0.00				
	Drinking	Nondrinker	30,382	12,451	13,060,663	0.95	1 (ref.)				1 (ref.)							
		Drinker	6419	2247	2,341,922	0.96	0.91	0.87	0.95	<0.0001	0.91	0.87	0.96	0.00				
	Physical activity	Average or health-promoting	9813	3862	4,699,042	0.82	1 (ref.)				1 (ref.)							
		Insufficient	26,988	10,836	10,703,543	1.01	1.02	0.99	1.06	0.25	1.02	0.98	1.06	0.39				

SS = survivorship stage. HR = hazard ratio, CI = confidential interval. Model 1: non-adjusted. Model 2: adjusted cancer type, health-related behavior, age at diagnosis, insurance premium quartile, medical history, BMI, systolic and diastolic blood pressure, fasting blood glucose, total cholesterol, high-density lipoprotein cholesterol, low-density lipoprotein cholesterol, and triglycerides. Model 3: adjusted cancer type, survivorship stage, age at diagnosis, insurance premium quartile, medical history, BMI, systolic and diastolic blood pressure, fasting blood glucose, total cholesterol, high-density lipoprotein cholesterol, low-density lipoprotein cholesterol, and triglycerides.

**Table 3:** Hazard ratios and 95% confidence intervals of depressive disorders based on cancer survivorship stage and health behaviors.

Variables			Participant (*n*)	Events (*n*)	Follow-Up Duration (Person-Years)	Incidence Rate (Per 1000 Person-Years)	Model 1 (Unadjusted)				Model 2				Model 3			
							HR	95% CI		*p*-Value	HR	95% CI		*p*-Value	HR	95% CI		*p*-Value
All cause depressive disorders			36,801	6168	5,703,419	1.08												
Cancer type		Breast	27,464	4346	3,904,167	1.11	1 (ref.)				1 (ref.)				1 (ref.)			
		Cervical	4873	911	1,038,435	0.88	1.14	1.06	1.22	0.00	1.04	0.97	1.12	0.26	1.14	1.06	1.23	0.00
		Ovarian	2329	506	389,607	1.30	1.40	1.27	1.53	<0.0001	1.40	1.28	1.54	<0.0001	1.40	1.27	1.53	<0.0001
		Endometrial	2135	405	371,210	1.09	1.18	1.07	1.31	0.00	1.17	1.06	1.30	0.00	1.18	1.07	1.31	0.00
SS		Acute SS	1198	227	42,045	5.40	3.14	2.74	3.61	<0.0001					2.99	2.60	3.42	<0.0001
		Extended SS	7052	1394	393,356	3.54	2.16	2.02	2.30	<0.0001					2.14	2.00	2.28	<0.0001
		Permanent SS	28,551	4547	5,268,018	0.86	1 (ref.)											
Health-related behavior	Smoking	Nonsmoker	35,012	5822	5,444,966	1.07	1 (ref.)				1 (ref.)							
		Former smoker	886	178	145,752	1.22	1.25	1.08	1.46	0.00	1.32	1.14	1.54	0.00				
		Current smoker	903	168	112,701	1.49	1.17	1.01	1.37	0.04	1.21	1.04	1.41	0.02				
	Drinking	Nondrinker	30,382	5204	4,869,048	1.07	1 (ref.)				1 (ref.)							
		Drinker	6419	964	834,371	1.16	0.89	0.82	0.95	0.00	0.96	0.89	1.03	0.26				
	Physical activity	Average or health-promoting	9813	1500	1,590,097	0.94	1 (ref.)				1 (ref.)							
		Insufficient	26,988	4668	4,113,322	1.13	1.14	1.08	1.21	<0.0001	1.09	1.02	1.15	0.01				

SS = survivorship stage, HR = hazard ratio, CI = confidential interval. Model 1: non-adjusted. Model 2: adjusted cancer type, health-related behavior, age at diagnosis, insurance premium quartile, medical history, BMI, systolic and diastolic blood pressure, fasting blood glucose, high-density lipoprotein cholesterol, low-density lipoprotein cholesterol, and triglycerides. Model 3: adjusted cancer type, survivorship stage, age at diagnosis, insurance premium quartile, medical history, BMI, systolic and diastolic blood pressure, fasting blood glucose, high-density lipoprotein cholesterol, low-density lipoprotein cholesterol, and triglycerides.

## Data Availability

Data were obtained from a third party and are not publicly available.
